# Effect of SARS-CoV-2 infection on human embryonic development and clinical outcomes: a retrospective cohort study

**DOI:** 10.1186/s12884-025-07205-y

**Published:** 2025-03-07

**Authors:** Li Tian, Yiting Sun, Miaomiao Jia

**Affiliations:** Center of Assisted Reproductive Technology, Northwest Women and Children’s Hospital, Xi’an, 710000 China

**Keywords:** COVID-19, Infection, Clinical outcome, Embryo

## Abstract

**Objective:**

To investigate the effect of SARS-CoV-2 infection on embryonic development and clinical outcomes.

**Methods:**

This retrospective analysis included 538 couples in December 2022. The couples were divided into two groups (COVID-19 group, *n* = 157; and non-COVID-19 [control] group, *n* = 381) according to whether one member of the couple had been infected with SARS-CoV-2 before oocyte retrieval. The general information, fertility rate, embryonic development and clinical outcomes were compared between the groups.

**Results:**

There were no significant differences in baseline characteristics between the two groups. The rates of fertility, good-quality embryos and blastocyst formation were similar between the two groups. The separate effects of male or female infection on embryonic development were further analyzed. The in vitro fertilization (IVF) fertilization rate was significant lower in the male COVID-19 group than in the control group (OR = 0.630, 95% CI = 0.510–0.776). In addition, the clinical pregnancy and live birth rate was significantly reduced in female patients who infected by SARS-CoV-2 compared to control group (OR = 0.018, 95% CI = 0.057–0.179).

**Conclusion:**

This study shows that infection before oocyte retrieval does not have a clear negative effect on embryo outcomes, such as the rates of normal fertilization, good-quality embryos and blastocyst formation. However, infection before oocyte retrieval has negative effects on clinical outcomes in female patients.

## Introduction

Severe acute respiratory syndrome coronavirus 2 (SARS-CoV-2) infection causes coronavirus disease 2019 (COVID-19). The SARS-CoV-2 virus enters target host cells through the angiotensin-converting enzyme 2 (ACE2) or cellular transmembrane serine protease 2 (TMPRSS2) route [1–3]. These route are found in the respiratory tract where the infection mostly occurs, but also in the reproductive tract.The existence of the ACE2 axis and ACE2 markers has been confirmed in testis, endometrium and all stages of ovary, including granulosa cells and follicular fluid [4–7]. In one study, SARS-CoV-2 RT-PCR-positive results were reported for 4 out of 38 (10.53%) cervical swabs samples from COVID-19-positive patients [8]. Another study detected SARS-CoV-2 in the semen of 4 out of 30 men, with acute infection [9]. Several studies indicate that SARS-CoV-2 virus might affect reproductive functions, through ACE2-mediated testis, ovarian and endometrial direct damage [10, 11].

Furthermore, some studies indicate that the body’s overactive immune response during COVID-19 may affect female and male reproductive function [12, 13]. In a cohort study of 123 COVID-19 male patients, an increasing antisperm IgA and IgG antibodies in the semen were observed, with significant seminiferous tubular injury, reduced Leydig cells, and mild lymphocytic inflammation [10]. Severe immune responses caused by SARS-CoV-2 may also impair the Hypothalamic-Pituitary-Ovarian (HPO) axis, leading to hormonal imbalance [14–16]. Significantly lower serum AMH levels were reported in 78 women with COVID-19 as compared with 151 healthy agematched controls [17]. This study also reported higher FSH, prolactin and testosterone levels in COVID-19 patients. This suggested disrupted ovarian reserve and function of infection woman.

Given these considerations, it is reasonable to suspect that COVID-19 may affect oocyte performance or early implantation. In a small case series including nine couples, the percentage of top-quality Day 3 embryos per fertilized oocyte was significantly lower after COVID-19, irrespective of whether the male or female partner was previously infected [18].

Generally, pregnant women were considered to be a high-risk group of developing SARS-CoV-2 infection. An analysis of 42 studies involving 438.548 pregnant women, comparing the ones with and without SARS-CoV-2 infection, found that pregnant women with SARS-CoV-2 infection were more likely to have preeclampsia, preterm birth, and increased perinatal mortality, as well as neonates with low birth weight [19].

There has been nationwide relaxation of controls regarding the COVID-19 epidemic since December 2022 in China. Fertility and in vitro fertilization (IVF) centers are receiving increasing numbers of infected patients.Nevertheless, the possible effects of COVID-19 on in vitro fertilization outcomes are largely unknown. Additionally, only a few studies have analyzed the effects of the ARS-CoV-2 virus on human oocytes and early-stage embryos. Therefore, this study aimed to evaluate the effect of SARS-CoV-2 infection on the outcomes of IVF treatment in fresh cycles. We established a prospective cohort study to assess the impact of COVID-19 on fertilization rate, embryo development and clinical pregnancy rates.

## Materials and methods

### Patients’ eligibility criteria

The ethical committee of Northwest Women’s and Children’s Hospital approved the study (LL-SC-SG-2022-007), and all the patients provided written informed consent.All methods were conducted in accordance with the relevant guidelines and regulations.

This retrospective single-center study included couples who were infected with SARS-CoV-2 and underwent fresh IVF treatment cycles (COVID-19 group) in the Reproductive Medical Center of Northwest Women’s and Children’s Hospital between December 1 ,2022 and December 31,2022.

To analyze the effect of SARS-CoV-2 infection on human embryonic development and clinical outcomes in the infectious stage, the maximal time from SARS-CoV-2 infection to treatment was defined as 1 week. SARS-CoV-2 infection was diagnosed by nucleic acid test of SARS-CoV-2. All enrolled patients were still positive when underwent treatment.Patients who underwent IVF treatment with no history of past infection in the same time period were recruited as the control group.

Patients were excluded from the study if they had planned to undergo oocyte or sperm donation, and social or medical freezing of oocytes. In addition, couples using thawed sperms or oocytes were excluded.

### Eligibility criteria

Nucleic acids from the SARS-CoV-2 virus were extracted from nasopharyngeal and throat swabs using an RNA virus kit (Daan Gene Co., Ltd., China). The reverse transcriptase-polymerase chain reaction assay was used to simultaneously detect two SARS-CoV-2 target genes (N gene and ORF1ab gene). The conditions for the amplification were 50 °C for 20 min and 95 °C for 10 min, followed by 40 cycles of denaturation at 95 °C for 15 s, and extension and fluorescence collection at 60 °C. Forty cycles were defined as the threshold value. Less than 40 cycles of denaturation indicated positivity for SARS-CoV-2.The sensitivity and specificity of this test were 79% and 99%,respectively.

### Ovarian stimulation, in vitro fertilization/intracytoplasmic sperm injection and embryo culture

Ovarian stimulation, in vitro fertilization/intracytoplasmic sperm injection, embryo culture and embryo biopsy were performed using standard protocols. Controlled ovarian hyperstimulation with gonadotropin started from day 2 of menses using recombinant follicle-stimulating hormone (Gonal F; Merck Serono, Italy) at an initial dose of 150–300 IU each day. A gonadotropin-releasing hormone antagonist was added when the follicular diameter reached 14 mm. When at least two follicles had reached 18 mm in size, human chorionic gonadotropin (Ovidrel^®^; Merck Serono, Germany) was injected as an ovulation trigger, and oocytes were retrieved 36 h later. Subsequently, oocytes were fertilized via either in vitro fertilization (IVF) or intracytoplasmic sperm injection (ICSI) following oocyte retrieval. The embryos were cultured in sequential media (G1-plus and G2-plus; Vitrolife) under 5% O_2_ and 6% CO_2_.

### Embryo transfer

Frozen–thawed embryos with an endometrial thickness of > 7 mm were transferred. Clinical pregnancy was defined as the presence of an intrauterine gestational sac with or without a fetal heartbeat on ultrasonography during the first trimester. Implantation rate was defined as the percentage of embryos that successfully implant into the uterine lining after being transferred to a woman’s uterus.The miscarriage rate is defined as the percentage of clinically recognized pregnancies that end in miscarriage.

### Statistical analysis

Data are presented as the mean ± standard deviation for continuous variables and as *n* (%) for categorical variables. Student’s t test and the Kruskal–Wallis rank test were used for parametric and nonparametric data, respectively. SPSS software version 22.0 (IBM SPSS) was used for statistical analysis and logistic regression analysis. A *P* value of < 0.05 was considered significant. A multivariate logistic regression analysis was used to examine the relationships between SARS-CoV-2 infection and clinical outcomes.And, embryo quality, female age, duration of infertility, endometrial thickness at embryo transfer, female BMI and bilateral AFC were adjusted for in the model.Odds ratios (ORs) and 95% confidence intervals (CIs) were calculated.

## Results

### Analysis of clinical characteristics

Due to SARS-CoV-2 infection, 25 patients who chose to freeze their oocytes were excluded from the study.A total of 157 SARS-CoV-2-infected couples and 381 non-infected couples who underwent IVF/ICSI treatment cycles were analyzed. The mean maternal age was 32.0 years (range, 22–50 years). The baseline characteristics were compared between the COVID-19 group and the control group. There were no differences in the patients’ age, basic luteinizing hormone concentrations, basal follicle stimulating hormone concentrations, infertility duration, body mass index(BMI) or antral follicle count between the two groups (Table 1). In addition, the total stimulation dose, the number of stimulation days and the insemination method were similar in the COVID-19 and control groups (Table 2).

**Table 1:** Epidemiologic characteristics COVID-19 and non-COVID-19 group

Parameter	COVID−19	non-COVID−19	*P*
No. of Cycles	157	381	
Female mean age (y, mean ± SD)	32.000 ± 4.466	32.360 ± 4.500	0.398
Duration of infertility (y, mean ± SD)	3.227 ± 2.220	3.029 ± 2.360	0.370
FSH, IU/L(Mean ± SD)	7.100 ± 2.573	7.480 ± 3.403	0.247
LH.IU/L(Mean ± SD)	4.706 ± 2.000	4.790 ± 3.274	0.777
No. of AFCs(Mean ± SD)	12.758 ± 7.144	11.675 ± 7.273	0.115
Female BMI, kg/m2(Mean ± SD)	22.687 ± 3.330	23.072 ± 3.490	0.239

***NOTE***: FSH = follicle-stimulating hormone; LH = Luteinizing hormone; BMI = body mass index; AFC = antral follicle count at baseline; *P < .05,two-sample Mann-Whitney test or PearsonX^2^ test

**Table 2:** Embryo outcomes in COVID-19 and non-COVID-19 group

Parameter	COVID−19	non-COVID−19	*P*
Fertilization type, *n* (%)			0.230
IVF	114(72.6)	295(77.4)	
ICSI	43(27.4)	86(22.6)	
Rescue ICSI	1(2.3)	3(3.4)	
Stimulation days(Mean ± SD)	10.350 ± 2.422	9.900 ± 2.617	0.451
Total dose of Gn, IU(Mean ± SD)	2401.194 ± 903.986	2325.893 ± 900.259	0.379
No. Of Retrieved oocytes	10.191 ± 5.730	9.580 ± 6.752	0.320
Normal fertilization rate, (%)	62.2(933/1509)	64.7(2217/3428)	0.113
Available embryos rate (%)*	86.7(814/939)*	83.8(1858/2217)	0.000
Good-quality embryos rate(%)	56.2(528/939)	59.5(1319/2217)	0.132
Blastocyst formation rate(%)	60.9(418/686)	61.7(977/1584)	0.293

Note *P < .05

### Effect of COVID-19 on laboratory outcomes in ART

The effect of COVID-19 on ART treatment at the infectious period was assessed. We found no differences in the number of oocyte retrieved, normal fertilization rate, good-quality embryos rate, or blastocyst formation rate between the two groups (Table 2). Notably, the available embryo rate in the COVID-19 group was higher than that in the control group (86.7% vs. 83.7%, *P* < .05). A available embryo was defined as four or more blastomeres on day 3, equally sized or slightly unequally sized blastomeres, and ≤ 15% fragmentation.

The separate effects of male or female infection on fertilization and embryo quality were further analyzed. In the group of men with COVID-19 whose wives had not been infected (male COVID-19 group) (*n* = 45), there was a similar available embryo rate and blastocyst formation rate to the control group (*n* = 381, Table 3). However, the normal fertilization rate was significantly lower in the male COVID-19 group than in the control group (54.9% vs. 64.7%), *P* < .005, OR = 0.666, 95% CI = 0.050–0.870) (Table 3). Furthermore, to analyze the effects of SARS-CoV-2 infection on fertilization in the male COVID-19 group, we divided the infected men into two subgroups according to the insemination method. We found that SARS-CoV-2 infection did not affect the fertilization rate in ICSI cycles, but significantly reduced the fertilization rate in IVF cycles compared with controls (52.5% vs. 63.5%, *P* < .005, OR = 0.630, 95% CI = 0.510–0.776) (Table 4). There were no differences in the rates of normal fertilization, available embryos or blastocyst formation between the group of women with COVID-19 whose husbands had not been infected (*n* = 49) and the control group (*n* = 381) (Table 4).

**Table 3:** Embryo outcomes in different subgroups of COVID-19 patients divided by sex

Sex of infected patient	Cycles (*n*)	Normal fertilization rate (%) OR(95%CI)	Available embryos rate (%)OR(95%CI)	Blastocyst formation rate(%) OR(95%CI)
Couples	63	66.1(372/563) 1.055(0.875–1.274)	86.3(321/372) 1.216(0.887–1.669)	58.3(155/266) 0.875(0.672–1.141)
Female	49	65.2(300/460) 1.024(0.835–1.256)	88.0(264/300) 1.422(0.986–2.050)	57.4(132/230) 0.845(0.638–1.120)
Male	45	54.9(267/468) 0.666(0.050–0.870)*	85.8(229/267) 1.168(0.814–1.677)	68.9(131/190) 1.379(0.988–1.906)
Control	381	64.7(2217/3428) 1	83.8(1858/2217) 1	61.8(977/1584) 1

Note *P < .05 Adjusted: Female age, male age, Duration of infertility, Female BMI

**Table 4:** The affect of COVID-19 patients on fertilization in different subgroups

Parameter	Cycles(*n*)	Normal fertilization rate (%)	OR(95%CI)	*P*值
IVF				
Couples	47	66.6(283/425)	0.874(0.705–1.085)	0.067
Female	32	63.9(209/327)	0.993(0.917–1.076)	0.382
Male	35	52.5(213/406)	0.630(0.510–0.776)^*^	0.002
Control	295	63.5(1741/2740)	1	
ICSI				
Couples	16	64.5(89/138)	0.788(0.537–1.155)	0.468
Female	17	68.4(91/133)	0.970(0.650–1.446)	0.579
Male	10	67.5(54/80)	0.929(0.576–1.522)	0.424
Control	86	69.2(476/688)	1	

Note *P < .05

### Effect of COVID-19 on clinical outcomes in ART

To analyze the effect of SARS-CoV-2 infection on clinical outcomes in the infectious stage, the maximal time from SARS-CoV-2 infection to embryo transfer was defined as 7 days. Forty of the 157 women in the COVID-19 group and 167 of the 381 women in the control group underwent fresh embryo transfer and were included in the pregnancy rate analysis.

Patients in the COVID-19 group and the control group had similar clinical characteristics. The clinical pregnancy rate was significantly lower in the COVID-19 group than in the control group (37.5% vs. 55.1%, *P* < .05) (Table 5). And the live birth rate were also reduced in female patients with COVID-19 compared to uninflected patients (25.0 vs. 49.1,*P* < .05)(Table 5).However, the early spontaneous abortion rate was similar in the two groups (Table 5).

**Table 5:** Clinical outcomes in COVID-19 and non-COVID-19 group

Parameter	COVID−19	non-COVID−19	*P*
Embryo transfer cycle (*n*)	40	167	
Female mean age (y, mean ± SD)	32.000 ± 4.076	32.090 ± 4.132	0.398
Endometrial thickness (mm)	12.340 ± 2.356	11.589 ± 2.269	0.060
No. of embryos transferred(mean ± SD)	1.300 ± 0.464	1.252 ± 0.435	0.160
No. of good-quality embryos transferred(mean ± SD)	0.850 ± 0.662	0.892 ± 0.549	0.565
Day of transfer	40	167	0.384
Day3	21(52.5)	75(44.9)	
Day5/6	19(47.5)	92(55.1)	
Clinical pregnancy(%)*	37.5(15/40)	58.1(97/167)	0.014
Early spontaneous abortion rate(%)	13.3(2/15)	11.3(11/97)	0.834
Live birth rate(%)*	25.0(10/40)	49.1(82/167)	0.001

Note *P < .05

The separate effects of male or female infection on the pregnancy rate were further analyzed. A multivariate logistic regression model of the pregnancy rate (Table 6) (adjusting female age, number of embryos transferred, day of transfer, embryo grade, and endometrial thickness, duration of infertility, female BMI) was performed to analyze the relationship between female SARS-CoV-2 infection and the pregnancy rate.

**Table 6:** Logistic regression model for clinical pregnancy rates in subgroup of patients

Parameter	Clinical pregnancy(%)	Adjusted OR(95%CI)	*P*
Cycles	207		
Couples	38.9(7/18)	0.576(0.207–1.603)	0.053
Female	28.6(4/14)	0.184(0.057–0.179)*	0.006
Male	50.0(4/8)	0.705(0.162–3.059)	0.437
Control	55.1(92/167)	1	

Note *P < .05

**Adjusted**: Female age, Number of embryos transferred, Day of transfer, Embryo grade, and Endometrial thickness, Duration of infertility, Female BMI

We found that the pregnancy rate was significantly lower in the female COVID-19 group than in the control group (28.6% vs. 55.1%, *P* < .05, OR = 0.184, 95% CI = 0.057–0.179) (Table 6). However, the pregnancy rate was similar in the group of SARS-CoV-2-infected men (*n* = 8) whose wives had not been infected and the control group (*n* = 157) (Table 6).

## Discussion

We analyzed the effect of COVID-19 on embryonic development and pregnancy at the acute infectious period of COVID-19(< 7 days).This study showed that SARS-CoV-2 reduced the normal fertilization rate of IVF cycles in male patients. However, SARS-CoV-2 had no effect on embryonic development. The clinical pregnancy rate was significantly lower in female patients with SARS-CoV-2 infection than in those without SARS-CoV-2 infection.

The co-expression of ACE2 and TMPRSS2 in gametes and fertilized eggs, as well as in blastocyst trophectoderm cells, suggests that oocytes and early-stage embryos may be affected by SARS-CoV-2 [20, 21]. However, our study showed that SARS-CoV-2 infection before oocyte retrieval had no negative effect on the embryo quality, including the rates of good-quality embryos, available embryos and blastocyst formation in male and female patients in the acute infectious period. Previous studies also support our conclusion that COVID-19 does not appear to be a risk factor in early human development [22, 23]. The mechanism of this phenomenon is still unknown. Some authors have hypothesized that SARS-CoV-2 viral RNA is not present in follicular fluid, oocytes and sperm [24, 25]. Given the uncertainties surrounding how infections might influence IVF outcomes, patients may feel hesitant to proceed with IVF treatments. In our study, 20 patients decided to halt their fertilization treatment and opt for freezing their oocytes.However, it may be unnecessary to cancel the treatment cycle in cases where concerns regarding recent SARS-CoV-2 infections do not significantly impact embryo quality.

Our findings reveal a significantly decreased fertilization rate among male patients.To evaluate possible mechanisms of this outcome, we stratified the male participants by fertilization method. Our study showed that COVID-19 significantly reduced the fertilization rate of male patients in IVF cycles but had no negative effect on the fertilization rate in ICSI cycles. The decreased sperm quality may explain the low fertilization rate in the COVID-19 group. In a cohort study of 123 COVID-19 male patients, an increasing antisperm IgA and IgG antibodies in the semen were observed, with significant seminiferous tubular injury, reduced Leydig cells, and mild lymphocytic inflammation [10]. Some studies also have reported a higher ICSI rate in patients with COVID-19, caused by reduced sperm quality, than in those without COVID-19 [25].

Further effects on implantation and pregnancy were evaluated in this study. We found that SARS-CoV-2 infection before embryo transfer had a significantly negative effect on pregnancy.Our data show that females who got infected as shortly as 7 days before their embryo transfer, show significantly reduced pregnancy rate and live birth. This result is not consistent with previous studies. Most previous studies showed a similar pregnancy rate in women with COVID-19 and controls [25–27]. Our study focus on the effect on acute infectious period of COVID-19 and all enrolled patients were still positive when underwent treatment.However, earlier research might include patients with varying time from infection to retrieval (range 0-2years).In addition, most of these studies chosen the control group based on medical history and did not undergo antibody testing or Nucleic acid test of SARS-CoV-2.Wang et al. [26], reported a compared pregnancy rate in infectious group and control group.They analyzed a group from Wuhan attending for IVF during a 10-month interval.The minimum interval between infection and ART treatments was 4 months and none of the patients had active COVID-19 when treated. ACE2 has been shown to be essential for human endometrial stromal cell decidualization, indicating that SARS-CoV-2 may be able to affect endometrial stromal cells and alter endometrial receptivity in women with COVID-19, thus possibly interfere with embryo implantation [12]. In addition to direct viral cell invasion, possible oxidative stress likely causes endometrial cellular damage in proximity to the acute infection.As reported by a previous study, over 90% (221/243) of patients infected by SARS-CoV-2 developed fever in the present study. Fever is a sign of acute immuneinflammatory responses, which may also affect endometrial receptivity [28].

Generally, pregnant women were considered to be a high-risk group of developing SARS-CoV-2 infection. An analysis of 42 studies involving 438.548 pregnant women, comparing the ones with and without SARS-CoV-2 infection, found that pregnant women with SARS-CoV-2 infection were more likely to have preeclampsia, preterm birth, and increased perinatal mortality, as well as neonates with low birth weight [29]. Another study shown that COVID-19 in pregnant women is associated with 11.1% rate of admission to ICU[]. This seemed to suggest that infection may have a negative impact on maternal-fetal outcomes.

In conclusion, there does not appear to be any evidence that SARS-CoV-2 infection during the vitro fertilization (IVF) treatment affect embryonic development.We currently do not recommend that cycle cancellation is necessary in those with SARS-CoV-2 infection while undergoing IVF/ICSI cycle.However, considering the negative effects on pregnancy outcomes in fresh transfer cycles, frozen embryo transfer (FET) cycles may be more suitable for women who have experienced infections.

The main limitation of the study is its retrospective study.The sample size of the study was relatively small, which may limit the statistical power to detect significant differences in fertilization rates and outcomes. Another caveat is the lack of sperm analyses, which, especially given the possible effect of SARS-CoV-2 infection on sperm parameters. Additionally, our study also lacks live birth and perinatal-neonatal outcomes. As such, we have designed a follow-up study to investigate long-term effects of COVID-19 on maternal and neonatal outcomes.

## Data Availability

The datasets generated and/or analysed during the current research are not publicly available as individual privacy could be compromised but are available from the corresponding author on reasonable request.
